# Incidence and predictors of loss to follow-up among women on option B+ PMTCT program in northwest Ethiopia. A retrospective follow-up study

**DOI:** 10.1371/journal.pone.0280546

**Published:** 2023-01-17

**Authors:** Habtamu Geremew, Awraris Wolde, Getachew Mullu Kassa

**Affiliations:** 1 College of Health Science, Oda Bultum University, Chiro, Ethiopia; 2 Department of Public Health, College of Health Science, Debre Markos University, Debre Markos, Ethiopia; International AIDS Vaccine Initiative, UNITED STATES

## Abstract

**Background:**

Loss to follow-up from lifelong antiretroviral therapy continued to be a major challenge affecting virtual elimination of mother-to-child transmission of human immunodeficiency virus, especially in Sub-Saharan Africa. Although there was a study conducted in Ethiopia, loss to follow-up was not clearly defined and some important variables were not addressed. Thus, this study was conducted to determine the incidence of loss to follow-up and its predictors among women on option B+ lifelong antiretroviral therapy program in Pawi district health facilities, northwest Ethiopia.

**Methods:**

An institutional-based retrospective follow-up study was conducted among 365 women who were enrolled for option B+ prevention of mother-to-child transmission service between June 2013 and March 2021 in Pawi district health facilities. A standard pretested checklist was used to extract data from all eligible women’s records. The Kaplan–Meier survival curve for estimating survival probability and Cox proportional hazards model to identify independent predictors of loss to follow-up were employed after checking for proportional hazards assumptions using STATA-14 statistical software.

**Result:**

The overall incidence of loss to follow-up was 12.04 (95% CI: 9.50, 15.20) per 1000 person-months of observation time. Residing outside the catchment area (adjusted hazard ratio (AHR): 3.08, 95% CI: 1.59, 5.98), lactating at enrollment (AHR: 2.43, 95% CI: 1.24, 4.77), living in a sero-discordant relationship (AHR: 2.5, 95% CI: 1.13, 5.53), lack of sero-status disclosure (AHR: 2.59, 95% CI: 1.15, 5.85), new enrollment to lifelong antiretroviral therapy (AHR: 2.07, 95% CI: 1.05, 4.11), and fair (AHR: 2.69, 95% CI: 1.2, 6.04) or poor (AHR: 5.78, 95% CI: 2.76, 12.12) antiretroviral drug adherence level were independent predictors of loss to follow-up.

**Conclusion:**

We found a higher incidence of loss to follow-up relative to previous studies in Ethiopia. Thus, strengthening adherence support interventions, and effective counseling on sero-status disclosure and male partner involvement are important to retain women in care.

## Introduction

Mother-to-Child Transmission (MTCT) of Human Immunodeficiency Virus (HIV) accounts for more than 90% of all new pediatric HIV infections [1]. It may occur in utero, during labor, delivery, and/or breastfeeding; without any intervention, the MTCT rate of HIV would range from 20% to 45% [1, 2]. Appropriate use of option B+ Prevention of Mother-to-Child Transmission (PMTCT) service is one of the four global strategies for eliminating MTCT of HIV along with primary prevention of HIV infection among childbearing women, Prevention of unwanted pregnancy among HIV-positive women, and providing appropriate treatment, care and support to mothers living with HIV and their families [2].

PMTCT programs are protecting hundreds of thousands of children each year from acquiring the virus, 1.4 million new HIV infections have been averted since 2010 among children in the world [3]. Even though option B+ PMTCT program has noticeably increased the accessibility and availability of antiretroviral therapy (ART), its effectiveness demands patient dedication and capability to adhere to the regimen and retain in care [4].

Loss to follow-up (LTFU) is still a major challenge affecting the effectiveness of PMTCT programs all over the world [5]. It is the most important cause of patient attrition from lifelong ART programs, especially in Sub-Saharan Africa [6, 7]. For instance, a retrospective cohort study in Uganda found an overall LTFU incidence of 30 per 1000 person-months of observation time [8]; furthermore, a study in Malawi revealed that the overall incidence of LTFU from option B+ PMTCT was 23.5 per 100 person-years of observation [9]. In Ethiopia, a scientific report indicated that the rate of LTFU from PMTCT care was 9 per 1000 person-months of observation time [10].

Retention in HIV care is a concern for all ART programs; however, stressors that facilitate LTFU are further complicated in pregnant and lactating women by significant biological, social and economic challenges associated with pregnancy and reproduction [2, 11, 12]. In addition to this, despite a higher rate of LTFU among people who start ART at or shortly after diagnosis [13, 14], option B+ PMTCT program recommends universal treatment of pregnant and lactating women starting at or shortly after diagnosis [15]; hence, making the program more vulnerable to LTFU.

Furthermore, LTFU was found to have a significant association with age, residence, educational status, pregnancy status at enrollment, stage of disease progression, disclosure of sero-status, partner HIV status, drug side effects, duration on ART and adherence to the lifelong ART regimen [8–10, 16, 17]. However, despite notable stigma and discrimination associated with the use of ready-to-use therapeutic foods [18], none of the previous studies have assessed its effect on LTFU. Besides, the effect of the level of health facility and residing within or outside of the catchment area was also not investigated.

The government of Ethiopia adopted option B+ PMTCT guidelines in 2013 as a means of eliminating mother-to-child transmission of HIV and ending the pandemic by 2030 [19]. Nevertheless, according to the UNAIDS report, the final vertical transmission rate including during breastfeeding in Ethiopia in 2019 was 17%, which is more than two-fold of the eastern and southern Africa’s regional average in the same year, 8% [20]. This variation in vertical transmission rate across countries is attributable to loss to follow-up from care and poor adherence to PMTCT services [6].

There was a study conducted to determine the incidence and predictors of LTFU among women on option B+ PMTCT program in western Ethiopia; however, despite using secondary data from the ministry of health registers and charts, the definition of LTFU was different from the ministry of health definition. Moreover, no recorded studies and/or documented reports were found in the current study area. Thus, this study was conducted to determine the incidence of LTFU and its predictors among women on option B+ PMTCT programs in Pawi district health facilities, northwest Ethiopia.

## Methods and materials

### Study design, area, and period

This institutional-based retrospective follow-up study was conducted in Pawi General Hospital and Felege Selam health center, which were the only health facilities that provide option B+ PMTCT service in Pawi District, Metekel Zone, Benishanguel Gumuz Region, northwest Ethiopia. The study was conducted retrospectively by using recorded data from June 2013 through March 2021, and the data were extracted from March 7 to March 31, 2021.

### Population

The source population was all women who were enrolled to option B+ PMTCT service in Pawi district health facilities. All women who were enrolled to option B+ PMTCT program in Pawi district health facilities between June 2013 and March 2021 as documented in the PMTCT register were our study population.

### Eligibility criteria

All women whose first appointment was at least one month before the date of data extraction were eligible to be included in the study so that all participants will have the opportunity to meet the definition of LTFU. However, Women with incomplete or missing clinical records regarding the variables of interest like date of enrollment to PMTCT, date event occurred, or women whose outcome/event was not recorded were excluded from the study.

### Sample size and sampling procedure

Records of all women who were enrolled to option B+ PMTCT program in Pawi district health facilities between June 2013 and March 2021 that fulfill the eligibility criteria were included. Yet, the adequacy of the sample size was verified by the Freedman method of proportional event allocation using the “stpower” command of STATA [21]. Consequently, the largest sample size obtained considering 95% confidence interval, 80% power, and 15% withdrawal probability was 306. Nevertheless, all records of women that fulfilled the eligibility criteria (365) were included to increase the power of the study.

### Data collection tool and procedures

The data were extracted using a previously validated standard data abstraction checklist adapted from previous literature [10, 14, 16]. The ART intake form, ART follow-up form, patient card and PMTCT register were reviewed to abstract the data by three Diploma Midwives who work in the respective facilities and they were supervised by a B.Sc. Midwife.

### Data quality control

To ensure data quality, the data extraction checklist was pretested to check its clarity, and one-day training was given to data collectors and the supervisor about the purpose of the study and how to abstract data from records. All selected data collectors and the supervisor were previously trained on the national PMTCT guideline. Besides, the extracted data were cautiously reviewed for completeness and consistency.

### Study variables and operational definition

The dependent variable of the study was loss to follow-up from option B+ PMTCT service, while independent variables include: socio-demographic and maternal characteristics of women at enrolment, clinical characteristics of women at enrollment and treatment-related characteristics of women. The event in the current study was LTFU, which was defined as missing an appointment for more than one month and not recorded as ‘dead’ or ‘transferred-out’ [19]. The ‘censored’ was defined as a woman who is recorded as dead, transferred-out, retained to ART, or receiving treatment when the study ended. Moreover, the survival time was defined as the time in months from enrolment to treatment under the option B+ PMTCT program to LTFU from the program.

### Data management and analyses

EpiData version-3.1 was used for data entry, and then it was exported to STATA version 14 for cleaning, coding and further statistical analysis. The survival experience of women was assessed using the Kaplan Meier survivor function, and survival experience between different groups of categorical independent variables was compared using the log-rank test. Both statistical and graphical methods were used to check for the Cox Proportional Hazard assumptions. Multicollinearity between independent variables was assessed using the variance inflation factor [22]. The likelihood ratio test was used to choose the best model [23].

Bi-variable Cox regression with a cutoff point of p-value less than 0.25 was used to select the potential candidate predictors for the full multivariable Cox proportional hazard model. The independent effect of predictors on the occurrence of LTFU was estimated by the Cox proportional hazard model and the association between predictor variables and LTFU was summarized using the Adjusted Hazard Ratio (AHR) with its respective 95% confidence interval (CI). Significant statistical association was declared at a p-value less than 0.05.

### Ethics issue

Ethical clearance was obtained from Debre Markos University College of Health Science Ethical review committee. Permission for data collection was granted from Pawi General Hospital and Felege Selam Health Center. Moreover, the confidentiality of extracted information was assured by not recording women’s personal identifiers.

## Results

### Socio-demographic and maternal characteristics of study participants

During the study period, a total of 422 women were enrolled for option B+ PMTCT care in the district. Of these, 365 women meeting the inclusion criteria were included in the final analysis, 12 were not eligible (whose first appointment was within a month from the date of data collection) while 45 records were incomplete in regard to variables of interest.

The age of women at enrollment ranged from 15 to 44 years with a median (interquartile range) age of 28 (25–32) years. One hundred fifty-six (42.7%) women belonged to the > = 30 years age group. The study revealed that 214(58.6%) study participants were urban residents, and 285(78.1%) were married. It also identified that most, 82.7% of the study participants were pregnant at enrollment (Table 1).

**Table 1:** Socio-demographic and maternal characteristics of women at enrolment to option B+ PMTCT program in Pawi district health facilities, northwest Ethiopia, June 2013- March 2021.

variable	Category	Frequency	Percentage
Age	< = 24	89	24.4
	25–29	120	32.9
	> = 30	156	42.7
Residence	Urban	214	58.6
	Rural	151	41.4
Women reside within the catchment area	Yes	302	82.7
	No	63	17.3
Marital status	Never married	26	7.1
	Married	285	78.1
	Divorced	41	11.2
	Widowed	13	3.6
Educational status	No formal education	141	38.6
	Primary	90	24.7
	Secondary	99	27.1
	Tertiary	35	9.6
Occupation	Housewife	236	64.7
	Governmental employee	45	12.3
	Farmer	32	8.8
	Merchant	20	5.5
	Student	14	3.8
	Daily worker	14	3.8
	Commercial sex worker	4	1.1
Religion	Orthodox	281	77.0
	Muslim	51	14.0
	Protestant	21	5.7
	Catholic	12	3.3
Pregnancy status at enrollment	Pregnant	302	82.7
	Lactating	63	17.3

### Clinical characteristics of study participants at enrollment

At enrollment, the majority (86.6%) of the women were classified as WHO Clinical Stage-Ι followed by stage-II 35(9.6%). Most, (74.3%) of the participants had a CD4 count of more than 350mg per dl of blood. Most, (315) women had disclosed their HIV sero-status; of which, 86.7% disclosed to their spouse followed by those who disclosed to their relatives 5.4% (Table 2).

**Table 2:** Baseline clinical characteristics of women on option B+ PMTCT program in Pawi district health facilities, northwest Ethiopia, June 2013- March 2021.

Variable	Category	Frequency	Percentage
Partner HIV status	Positive	174	47.7
	Negative	77	21.1
	Unknown	114	31.2
WHO stage at enrollment	One	316	86.6
	Two	35	9.6
	Three	11	3.0
	Four	3	0.8
Anemia	Non-anemic	309	84.7
	Anemic	56	15.3
Weight at enrollment	>55 kg	193	52.9
	< = 55 kg	172	47.1
Nutritional status	Healthy	250	68.5
	MAM	92	25.2
	SAM	23	6.3
CD4	>350	271	74.3
	< = 350	72	19.7
	Not determined	22	6.0
Viral load	Determined	209	57.3
	Not determined	156	42.7
TB screening status	Negative	361	98.9
	Positive	4	1.1
Opportunistic infection	Yes	54	14.8
	No	311	85.2
Disclosure status	Yes	315	86.3
	No	50	13.7
To whom did she disclose	Spouse	273	86.7
	Relative	17	5.4
	Siblings	14	4.4
	Friends	7	2.2
	Parents	3	1.0
	Own children	1	0.3
Functional status	Working	354	97.0
	Ambulatory	8	2.2
	Bedridden	3	0.8

### Treatment-related characteristics of study participants

More than half (53.2%) of participants didn’t start ART on the same day of diagnosis. Most, (66.3%) of women were on ART before enrollment to PMTCT whereas the remaining were newly enrolled. The predominant regimens initially prescribed were a combination therapy TDF/3TC/EFV for 224(61.3%) participants, followed by AZT/3TC/NVP for 62(17.0%). The majority, 86.0% of women attended treatment at the hospital. Two hundred eighty-six (78.4%) had no history of cotrimoxazole prevention therapy (CPT) while most, 93.2% of the women took isoniazid (INH) (Table 3).

**Table 3:** Treatment-related characteristics of women on option B+ PMTCT program in Pawi district health facilities, northwest Ethiopia, June 2013- March 2021.

Variable	Category	Frequency	Percentage
Time of ART initiation	Same day	171	46.8
	Later	194	53.2
Enrollment type to PMTCT	From ART	242	66.3
	New	123	33.7
Facility treatment attended	Hospital	314	86.0
	Health center	51	14.0
ART adherence level	Good	308	84.4
	Fair	30	8.2
	Poor	27	7.4
Initial ART regimen	AZT+3TC + NVP	62	17.0
	AZT+3TC + EFV	29	8.0
	TDF+3TC+EFV	224	61.3
	TDF+FTC+NVP	40	11.0
	TDF+3TC+DTG	10	2.7
Change in regimen	Yes	70	19.2
	No	295	80.8
Reason for regimen change	New drug	58	82.8
	Failure	7	10.0
	Toxicity	3	4.3
	Pregnancy	2	2.9
Recent regimen	AZT+3TC + NVP	33	9.0
	AZT+3TC + EFV	19	5.2
	TDF+3TC+EFV	244	66.9
	TDF+FTC+NVP	28	7.7
	TDF+3TC+DTG	34	9.3
	TDF+3TC+ATV/r	3	0.8
	TDF+3TC+LPV/r	4	1.1
Drug side effect	Yes	26	7.1
	No	339	92.9
History of TB treatment	Yes	5	1.4
	No	360	98.6
Experienced nutritional supplements	Yes	28	7.7
	No	337	92.3
Maternal CPT	Yes	79	21.6
	No	286	78.4
Maternal INH	Yes	340	93.2
	No	25	6.8

### Survival status of women

A total of 365 women were followed for a median follow-up time of 16.27 months. During the follow-up time, a total of 5895.27 person-months time risk was observed with a minimum and maximum follow-up time of 1.20 and 35.77 months, respectively. Totally, 71(19.5%) women were LTFU during the follow-up period. The majority, 173(47.4%) of the women completed the program and transferred to ART follow-up, while 34(9.3%) of them were transferred out to other health institutions before completing the program. By the end of the study, 85(23.3%) women were receiving care in the PMTCT program, while 2(0.5%) were dead.

### Incidence and time to loss to follow-up

**Fig 1:**
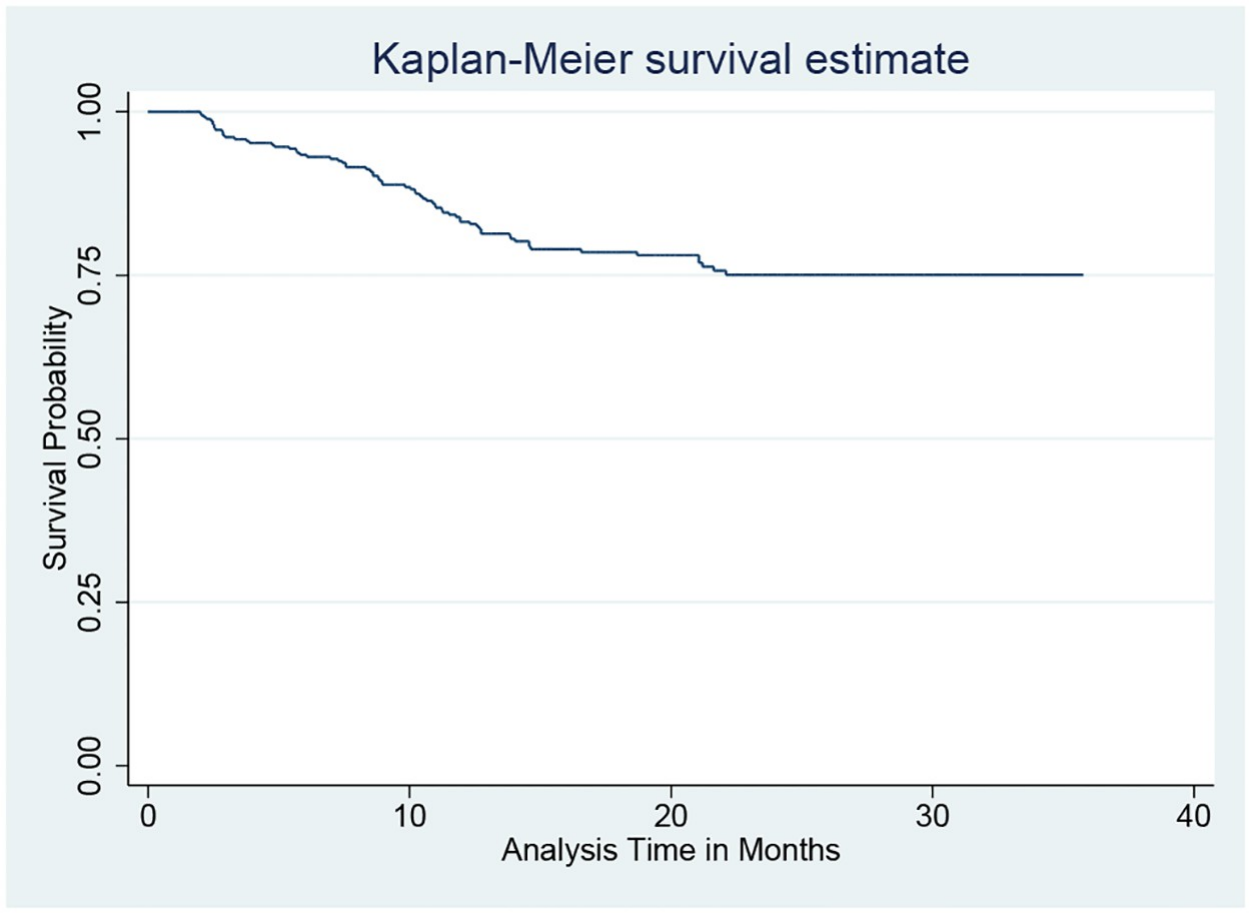
Overall Kaplan–Meier survival function estimates of HIV-positive women on option B+ PMTCT program in Pawi district health facilities, northwest Ethiopia, June 2013- March 2021.

The overall incidence of LTFU was 12.04 (95% CI: 9.50, 15.20) per 1000 person-months of observation time. The study also found that the restricted mean survival time of women was 29.43 (95% CI: 28.13, 30.73) months. The highest incidence of LTFU was observed at the 3rd month of follow-up (37/1000 person-months observations) (Fig 1).

### Predictors of loss to follow-up

Of twenty variables considered for multi-variable cox, the final model with 14 variables was selected using the likelihood ratio test. According to the multivariable Cox regression analysis, six of the predictors (residing within the catchment area, pregnancy status at enrollment, partner HIV status, disclosure of HIV status, new enrollment to PMTCT care, and level of ART adherence) were found to be independent predictors of LTFU. Women who reside out of the catchment area had a 3.08 (AHR: 3.08, 95% CI: 1.59, 5.98) times higher risk of LTFU when compared to women who reside within the catchment area. The hazard of LTFU was 2.43 (AHR: 2.43, 95% CI: 1.24, 4.77) times higher among lactating women as compared to women who were pregnant at enrollment. Likewise, the risk of LTFU was 2.5 (AHR: 2.5, 95% CI: 1.13, 5.53) times higher among women whose partners were HIV negative when compared to women with HIV positive partner.

This study also showed that women who had not disclosed their HIV sero-status had a 2.59 (AHR: 2.59, 95% CI: 1.15, 5.85) times higher risk of LTFU than those women who had disclosed their HIV sero-status. Women who were newly enrolled to lifelong ART were having 2.07 (AHR: 2.07, 95% CI: 1.05, 4.11) times higher hazard of LTFU than women who were previously on ART. Moreover, the risk of LTFU was higher among women who had fair 2.69 (AHR: 2.69, 95% CI: 1.2, 6.04) or poor 5.78 (AHR: 5.78, 95% CI: 2.76, 12.12) level of ART drug adherence as compared to women who had a good level of ART adherence (Table 4).

**Table 4:** Predictors of LTFU among women on Option B+ PMTCT program in Pawi district health facilities, northwest Ethiopia, June 2013- March 2021.

Variable	Category	Survival Status		CHR	AHR (95%CI)
		LTFU, No(%)	Censored, No(%)		
Reside in catchment	Yes	35 (11.6)	267 (88.4)	1	1
	No	36 (57.1)	27 (42.9)	7.07	3.08 (1.59, 5.98)*
Pregnancy status	Pregnant	50 (16.6)	252 (83.4)	1	1
	Lactating	21 (33.3)	42 (66.7)	3.2	2.43 (1.24, 4.77)*
Partner	Positive	13 (7.5)	161 (92.5)	1	1
HIV status	Negative	17 (22.1)	60 (77.9)	3.34	2.5 (1.13, 5.53)*
	Unknown	41 (36.0)	73 (64.0)	6.03	1.05 (0.44, 2.49)
Disclosure	Yes	36 (11.4)	279 (88.6)	1	1
	No	35 (70.0)	15 (30.0)	8.84	2.59 (1.15, 5.85)*
Enrollment type	From ART	21 (8.7)	221 (91.3)	1	1
	New	50 (40.7)	73 (59.3)	6.56	2.07 (1.05, 4.11)*
Initial regimen	AZT+3TC + NVP	19 (30.7)	43 (69.3)	1.87	1.39 (0.63, 3.07)
	AZT+3TC + EFV	8 (27.6)	21 (72.4)	1.54	1.2 (0.44, 3.27)
	TDF+3TC+EFV	35 (15.6)	189 (84.4)	1	1
	Others^#^	9 (18.0)	41 (82.0)	1.04	0.62 (0.24, 1.59)
Regimen change	Yes	21 (30.0)	49 (70.0)	1.58	1.43 (0.69, 3)
	No	50 (17.0)	245 (83.0)	1	1
Age group	< = 24	37 (41.6)	52 (58.4)	3.94	1.87 (0.94, 3.72)
	25–29	15 (12.5)	105 (87.5)	1.04	0.59 (0.25, 1.36)
	> = 30	19 (12.2)	137 (87.8)	1	1
Level of education	No formal education	34 (24.1)	107 (75.9)	1.49	1.33 (0.62, 2.86)
	Primary	12 (13.3)	78 (86.7)	0.73	0.66 (0.27, 1.62)
	Secondary & above	25 (18.7)	109 (81.3)	1	1
Occupation	Housewife	28 (11.9)	208 (88.1)	1	1
	Farmer	10 (31.3)	22 (68.7)	3.43	1.85 (0.76, 4.49)
	Gov’t employee	11 (24.4)	34 (75.6)	2.05	1.33 (0.52, 3.44)
	Merchant	11 (55.0)	9 (45.0)	6.05	1.76 (0.66, 4.69)
	Others^+^	11 (34.4)	21 (65.6)	3.53	2.4 (0.94, 5.68)
CD4	>350	45 (16.6)	226 (83.4)	0.88	0.9 (0.43, 1.88)
	< = 350	14 (19.4)	58 (80.6)	1	1
	Not determined	12 (54.5)	10 (45.5)	6.14	2.19 (0.85, 5.68)
Viral load	Determined	35 (16.7)	174 (83.3)	1	1
	Not determined	36 (23.1)	120 (76.9)	1.49	1.41 (0.78, 2.56)
Time of initiation	Same day	44 (25.7)	127 (74.3)	2.17	1.43 (0.78, 2.66)
	Later	27 (13.9)	167 (86.1)	1	1
Adherence level	Good	35 (11.4)	273 (88.6)	1	1
	Fair	16 (53.3)	14 (46.7)	6.94	2.69 (1.2, 6.04)*
	Poor	20 (74.1)	7 (25.9)	15.28	5.78 (2.76, 12.12)*

Key; AHR-adjusted hazard ratio, CHR-crude hazard ratio, CI-confidence interval, Others^#^ -TDF+3TC+DTG or TDF+FTC+NVP, Other^+^-daily laborer, commercial sex worker or students,

*- significant at p-value < 0.05.

## Discussion

This study was conducted to determine the incidence and predictors of LTFU among women on option B+ PMTCT program in Pawi district health facilities. The incidence of LTFU in our study was higher than has been reported by previous studies done in Ethiopia [10, 14, 16], This might be due to variations in the definition of LTFU. The fact that the current study was conducted in one of the underserved regions of the country, where there are limited infrastructures and resources could also be another possible elucidation for the variation. This connotes that interventions targeting these groups of women could result in an improved effectiveness of PMTCT programs.

There was also a variation in the incidence rate of LTFU in this study relative to earlier reports from other countries. For instance, the present LTFU incidence was higher than a report from Malawi 23.5 per 1000 person-years (this is equivalent to 1.96 per 1000 person-months) [9]. This could be partly due to the difference in the time of program introduction. Malawi was the first country to start option B+ PMTCT service and introduced the program in July 2011 which is even before WHO updated the guideline in April 2012 [24], this might result in an established experience with the program.

On the other hand, our LTFU incidence estimate was lower than findings from Tanzania 76 per 1000 person-months [17], and Uganda 30 per 1000 person-months [8]. This might be due to the difference in characteristics of study participants, which all the women included in the Tanzania report were newly diagnosed (not on ART before) and 92% of participants in the Uganda study were from rural areas. This reasoning is verified by previous studies which revealed that newly enrolled women and women from rural areas had a higher risk of LTFU [10, 14, 25].

The highest rate of LTFU incidence was observed at the 3rd month in the present study. This is in line with previous studies which found higher early LTFU rates [16, 26]. Such a higher rate of LTFU at the early stage of lifelong ART, particularly in the first three months of treatment, could be attributed to early adverse drug reactions and drug hypersensitivity [2].

The present study also identified different predictors of LTFU. Accordingly, women who reside out of the catchment area were 3.08 times more likely to be LTFU as compared to those who reside within the catchment area. This could be partly because residing out of the catchment area might add transportation costs and hence competing priorities of other basic needs like feeding their families rather than attending PMTCT programs. Consequently, measures like appointment spacing conjugated with proper counseling are important to retain such women. Likewise, lactating women were having 2.43 times higher risk of LTFU than women who initiated the program during pregnancy. This association was also clearly demonstrated by previous studies [25–27]. This might be due to the mother’s perception that the child is already infected by the virus during gestation and/or delivery [28].

The risk of LTFU was 2.5 times higher among women in an HIV sero-discordant relationship when compared to those who are in an HIV sero-concordant relationship. This was in line with a previous study [29], and might be related to the opposition and lack of support from their husbands. Hence, partner involvement in PMTCT is crucial for successful program implementation. Consistent with previous studies, women who had not disclosed their HIV sero-status had a 2.59 times higher risk of LTFU than those women who had disclosed their status [10, 30, 31]. This might be related to women who had not disclosed their status might travel out of their usual residence for ARV refill to avoid identification hence increasing physical barriers. Newly enrolled women were having a higher risk of LTFU than those women who were previously on ART. This might be due to poor knowledge about the purpose and possible side effects of ART among newly enrolled women.

Women who had fair or poor ART drug adherence were having higher hazard of LTFU as compared to women who had a good level of ART adherence. This finding was also supported by previous findings in Ethiopia [10] and Malawi [32]. This could be due to poor knowledge and lack of effective counseling on the importance of adherence to ARVs and PMTCT follow-up which may lead to stopping/missing the schedule of ART treatment. This justification is strengthened by previous studies which found that mothers who know ART drugs work had 65% lower risk of LTFU [28]. Besides, not understanding the first counseling session was among the reasons for LTFU [9]. Thus, proper patient education and counseling on the importance of adherence to PMTCT service could have a tremendous impact on the retention of women in lifelong ART care.

The current study is not without limitations. As it was based on secondary data, potentially important variables like socioeconomic status, distance from the PMTCT center, and waiting time within the clinic were not obtained from the records. Besides, the outcome of women who were recorded as loss to follow-up was not ascertained; hence, they might actually be dead or silently transferred to other facilities.

## Conclusion

This study indicated a higher incidence of LTFU among women on option B+ PMTCT program relative to previous studies in Ethiopia. Thus, strengthening adherence support interventions, and effective counseling on sero-status disclosure and male partner involvement are important to retain women in care. Further prospective and qualitative studies are warranted.

## Supporting information

S1 Dataset (DTA)
